# Microcatheter-Assisted Protection of the Posterior Inferior Cerebellar Artery During Parent Artery Sacrifice of a Vertebral Artery Dissecting Aneurysm

**DOI:** 10.7759/cureus.15773

**Published:** 2021-06-20

**Authors:** Daniel Loh, Francis A Basilio, Leanne Tan, Julian Han, Wickly Lee

**Affiliations:** 1 Neurosurgery, National Neuroscience Institute, Singapore, SGP; 2 Neuroradiology, National Neuroscience Institute, Singapore, SGP

**Keywords:** vertebral dissecting aneurysm, parent artery sacrifice, branch artery preservation, microcatheter protection, posterior inferior cerebellar artery

## Abstract

Branch vessel occlusion is a major cause of stroke in parent artery sacrifice (PAS) for vertebral artery dissecting aneurysms (VADA). There is now an increasing trend towards preservation of branch vessels during PAS. Stents are commonly employed to achieve this but bring with it the attendant risks of future thrombosis and lifelong antiplatelet use. Although a microcatheter protection technique has been utilised in branch artery protection of wide-necked saccular aneurysms, it has rarely been described in PAS for VADAs. We describe the use of a dual microcatheter technique in the protection and remodelling of the posterior inferior cerebellar artery (PICA) during PAS of the vertebral artery, which also served as a temporary scaffold to support placement of the coils during the embolisation process.

## Introduction

Parent artery sacrifice (PAS) for vertebral artery dissecting aneurysms (VADAs) is a well-established technique [1]; the primary aim is permanent isolation and occlusion of the diseased segment to prevent secondary dissection, thrombo-embolic and haemorrhagic events. Besides the inherent stroke risk associated with PAS, the other major cause of morbidity results from occlusion of branch vessels, notably the posterior inferior cerebellar artery (PICA), arising from or immediately adjacent to the diseased segment. There is an evolving trend towards preservation of branch arteries, which is commonly achieved with the use of stents or flow diverters [2-4]. Although PICA preservation without the use of adjuncts during vertebral artery occlusion has been reported [5], definitive protection of the PICA is still preferable. 

A microcatheter protection technique is one of the described assisted techniques used to preserve parent or branch arteries in endovascular embolisation of wide-necked saccular aneurysm; however, this technique is rarely employed in embolisation of VADAs. We describe the technique of microcatheter protection of the PICA during PAS of a VADA resulting in complete occlusion of the vertebral artery while simultaneously preserving the adjacent PICA, without the use of a stent or flow diverter. 

## Technical report

A 57-year-old woman presented with a World Federation of Neurosurgical Societies Grade III, Modified Fisher Grade IV subarachnoid haemorrhage. Computed tomographic angiography (CTA) showed a right VADA affecting the proximal V4 segment.

**Figure 1:**
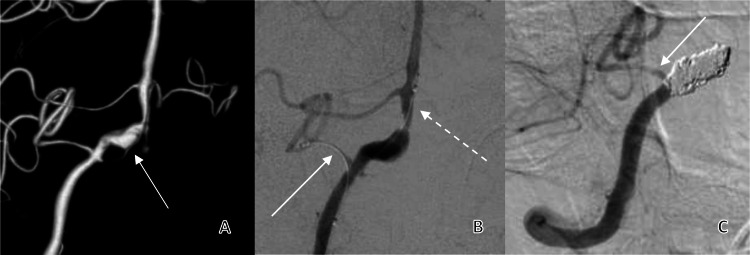
Catheter embolisation of the right vertebral artery dissecting aneurysm with micro-catheter protection of the posterior inferior cerebellar artery A. Three-dimensional rotational angiography of the right vertebral artery demonstrating the dissecting aneurysm just distal to the posterior inferior cerebellar artery origin (white arrow). B. Temporary cannulation of the right posterior inferior cerebellar artery using the 2.3-Fr Prowler select microcatheter (solid arrow) and the coiling 1.7-Fr Echelon-10 microcatheter (dashed arrow). C. Post-embolisation angiography showing full occlusion of the dissecting aneurysm of the vertebral artery segment with preservation of the posterior inferior cerebellar artery (white arrow).

Catheter digital subtraction angiogram confirmed a 5.3 x 11.5 mm (diameter x length) dissecting aneurysm (Figure 1A) involving the right vertebral artery just distal to the PICA origin. Contralateral angiography demonstrated good calibre of the left vertebral artery.

A right transfemoral approach was used to insert a 6-Fr guiding catheter into the cervical vertebral artery. After choosing the best working projection by using a biplane angiography unit (GE innova 3131-IQ), a roadmapping technique was done.

A 1.7-Fr microcatheter (Echelon-10, Medtronic) was advanced to the right vertebral artery aneurysm. A 2.3-Fr microcatheter (Prowler select plus, Codman Neurovascular) was then inserted in tandem, and was used to cannulate the PICA (Figure 1B). Under systemic heparinization, detachable coils (Target 360 coils, Stryker; Axium Prime coils, Medtronic) were subsequently deployed within the aneurysm. The microcatheter within the PICA was utilized as a temporary remodelling scaffold to provide manual stabilization of the coils during occlusion of the right vertebral artery, preventing the coils from occluding the PICA origin. The microcatheters were then removed. Post-embolization angiogram showed complete occlusion of the aneurysm and right vertebral artery with preservation of the PICA (Figure 1C).

**Figure 2:**
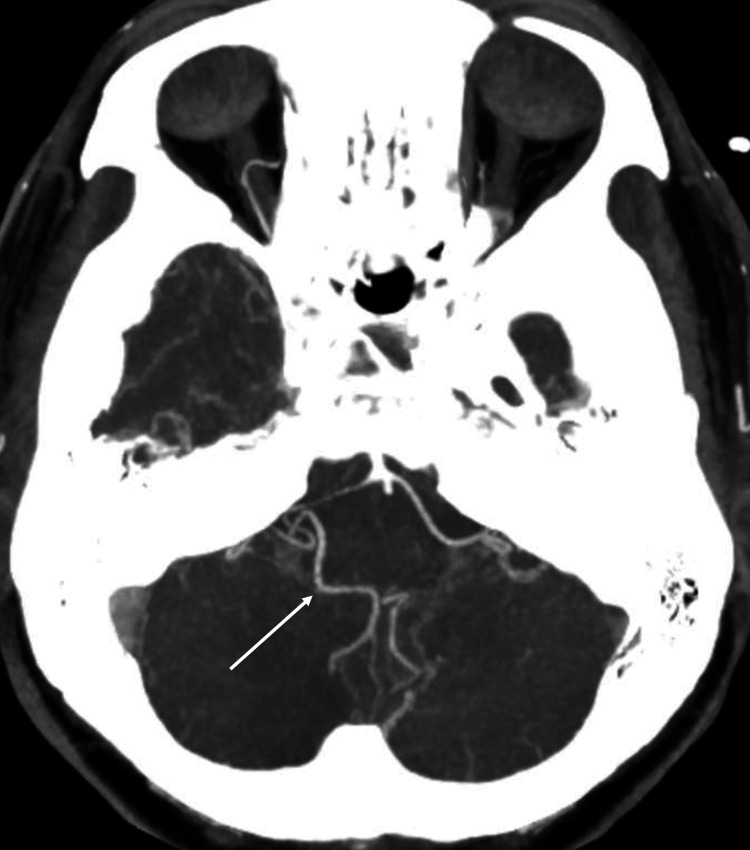
Post-procedure computed tomography angiography demonstrating continued preservation of the posterior inferior cerebellar artery (white arrow)

Follow-up CTA (Figure 2) showed continued patency of the right PICA. The patient recovered well with a mild right dysmetria and was transferred to a tertiary rehabilitation centre for continued care.

## Discussion

The technique of microcatheter protection of the PICA has been described in angioplasty of atherosclerotic intracranial lesions [6] and in three cases of vertebral saccular aneurysms incorporating a branch vessel [7,8]. However, after a thorough literature search, we found no prior description of the technique in the complete coiling of a VADA and reconstruction of the PICA.

In our case, preservation of the PICA to minimize complications of side branch occlusion was important because of its larger-than-normal calibre and presumed larger vascular territory. Moreover, there was angiographic absence of collateral supply either from an ipsilateral AICA-PICA complex or contralateral bihemispheric PICA disposition. Its larger-than-normal calibre also facilitated temporary cannulation with a microcatheter to enable reconstruction of the PICA into an end vessel while the parent artery was being occluded. 

This technique avoids the use of stents, reducing costs and obviating the need for periprocedural and lifelong antiplatelet therapy. The risk of delayed stent occlusion in the PICA is also eliminated. Cannulation of the PICA also serves as a means of remodelling for the reconstruction of the PICA origin and provides mechanical stabilisation to enable better packing of the coils in the dissected segment, reducing the risk of recurrence. This is achievable even with the higher coil mass needed for parent artery occlusion.

## Conclusions

Branch artery protection without the use of stents is feasible in selected cases where the vessel origin is just adjacent to (but not within) the diseased segment. This ameliorates the morbidity of procedure-related infarcts while eliminating stent-related complications. This technique is also applicable in VADAs proximal to the PICA origin where the microcatheter can be introduced via the contralateral vertebral artery.
